# Electronic Skins for Advanced Wound Healing: Biomimetic Thermoregulation and Bioelectrically Active Systems

**DOI:** 10.3390/polym18050586

**Published:** 2026-02-27

**Authors:** Nianhao Xue, Wenhao Guan, Tanghao Xia, Kexue Sun

**Affiliations:** 1 College of Electronic and Optical Engineering & College of Flexible Electronics (Future Technology), Nanjing University of Posts and Telecommunications, Nanjing 210023, China; b23020328@njupt.edu.cn (N.X.);; 2 College of Electrical and Automation Engineering, Nanjing Normal University, Nanjing 210023, China; 3 Nation-Local Joint Project Engineering Lab of RF Integration & Micropackage, Nanjing 210023, China

**Keywords:** electronic skin, wound healing, thermoregulation, bioelectrical stimulation, self-healing polymers

## Abstract

Urgent demand for wound healing treatments has driven rapid advancement in electronic skin technology. As a promising wound healing approach, electronic skin offers advantages such as flexible conformability, autonomous sensing, and intelligent regulation. However, mainstream electronic healing patches face significant challenges in complex wound applications, including insufficient coordination, delayed response, limited healing efficiency, and inadequate feedback. Therefore, developing innovative wound healing technologies that integrate high efficiency, multi-module drive, and closed-loop feedback is imperative. The advanced development of electronic skin for wound healing is urgently needed to be systematically reviewed. Here, first, the structural innovations and design strategies for biomimetic thermotherapeutic electronic skins based on thermoelectric polymer composites and interactive temperature biomimetic regulation are summarized. Subsequently, several emerging bioelectrically active electronic skins are reviewed, including drug-delivery electronic skins, multifunctional hydrogel-integrated electronic skins, and photoelectric synergistic stimulation electronic skins, along with an analysis of their advanced designs and innovative advantages. Last but not least, potential challenges facing the future development of electronic skin are explored. Practical solutions are proposed for advancing low-cost, clinically applicable, and scalable electronic skin development, aiming to drive breakthrough progress in therapeutic wound healing.

## 1. Introduction

The development of novel electronic patches for wound healing has become particularly urgent against the backdrop of accelerating global aging and a sharp rise in wound incidence, driven by surging prevalence rates of chronic diseases and metabolic disorders [1,2,3]. As an innovative electronic patch technology, electronic skin offers a practical solution for the efficient treatment of complex wounds. Currently, mainstream wound healing methods on the market can be summarized as applying passive interventions to wounds [4], primarily encompassing mechanical barrier healing, implantable electrical stimulation devices, and growth factor release therapies [5,6,7]. Among these, electronic patches, which combine the mechanical care of traditional physical barriers with integrated platform control capabilities, have gradually become a focal point for researchers [8,9,10].

Recent epidemiological studies indicate that chronic non-healing wounds now affect 1–2% of the global population, equating to hundreds of millions of patients [11]. Inhibiting wound expansion and promoting healing through electronic patch technology has become a critical issue for advancing health development. Currently, commercially available electronic healing patches primarily include passive sensing patches, physical stimulation patches, and simple controlled-release drug patches [12,13,14]. The passive sensing electronic patch can detect information such as temperature, humidity, and pH near the wound, reflecting the objective wound condition [15,16,17]. The physical stimulation electronic patch delivers controllable stimuli such as microcurrent, electric fields, and light stimulation, accelerating the natural wound healing process through external factors [18]. Additionally, the simple controlled-release drug patch, utilizing a growth factor release platform, can efficiently modulate fibroblast-directed migration and ordered collagen deposition, accelerating the generation of regenerative tissue at the wound site [19,20,21].

However, passive dressings lack integrated sensing and treatment coordination. Without active monitoring, their prolonged adhesion can cause peripheral tissue damage and significantly delay healing [22,23]. Rigid electrotherapy electrodes generate physical stimulation, but their mechanical mismatch with tissue can induce secondary injury. Furthermore, these electrodes generally suffer from insufficient signal coupling efficiency [24]. The lack of autonomous sensing and targeted delivery capabilities in growth factor carriers may trigger overload issues such as redundant repair and excessive angiogenesis [25,26]. More critically, the aforementioned traditional technologies exhibit relatively fragmented functionality and lack closed-loop feedback and intelligent regulation, limiting their universal application in complex wound management [22,27,28]. Moreover, wound healing itself represents a highly complex physiological process spanning inflammation regulation, cellular angiogenesis, and tissue remodeling. Its dynamic changes reflect various pathological states such as infection, ischemia, and inflammatory imbalance [29,30,31,32].

Therefore, against the backdrop of a global surge in prevalence, the technical demands for wound healing are progressively increasing, creating an urgent need for innovative technologies based on electronic patches [33,34]. Meanwhile, electronic skin integrates sensing, actuation, and self-regulation capabilities into a single unit, featuring high perceptual dimensions, strong intelligent feedback loops, and flexible scalability. Through device innovation and multimodal platform development, electronic skin can overcome the limitations of traditional wound healing solutions [35,36,37]. Although electronic skin has become a significant frontier in current research, systematic summaries of its innovative technological developments and potential applications remain limited. Therefore, conducting a more comprehensive review is particularly urgent for deepening understanding and further optimization.

**Figure 1:**
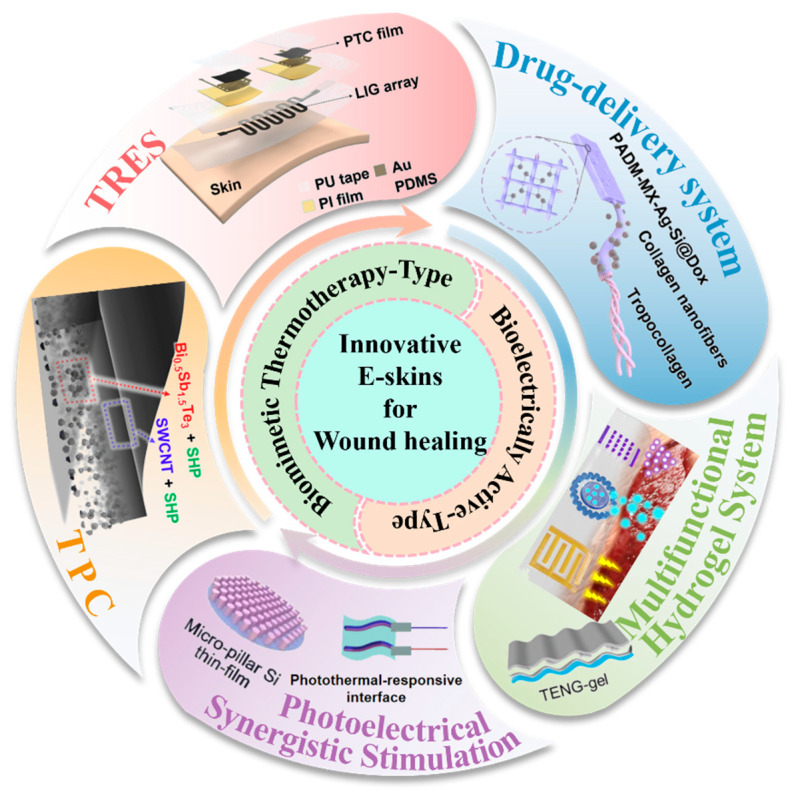
Summary diagram of innovative electronic skins for wound healing.

This review highlights recent advances and breakthrough innovations in electronic skin design for wound healing applications. As shown in Figure 1, we first introduce a bionic heat-patch electronic skin based on thermoelectric polymer composites with interactive temperature-mimetic regulation capabilities, detailing the innovative structure and mechanism of TPC and TRES. Subsequently, we systematically summarize bioelectrically active electronic skins, pioneering an analysis of an innovative drug-delivery electronic skin technology along with its application data. We then review two electronic skins integrating multifunctional hydrogels. Building upon single electrical stimulation, we delve into strategies and potential applications of electronic skins based on photoelectric synergistic stimulation. Finally, after summarizing the review’s content, we engage in a critical discussion of existing research and outline future directions for electronic skin development.

Our objective is to provide novel and forward-looking insights to drive continuous innovation and advancement in the field of wound healing.

## 2. Ideal Conditions for Effective Wound Healing

Ideally, effective wound healing relies on a repair environment that is both dynamically adjustable and relatively stable. The initial step in wound management is typically to control the inflammatory response [38]. The early phase of inflammation is autonomously activated to efficiently clear pathogens, necrotic tissue, and metabolic waste [39,40]. It is also important to prevent secondary wound damage caused by persistent inflammatory responses, such as downregulating the expression of inflammatory factors like TNF-α, thereby maintaining inflammation at an ideal, transient state [41,42,43]. Moreover, rapid vascularization, along with efficient cell migration and proliferation, constitutes an ideal condition for wound healing. These processes are essential because healthy blood vessels perform vital functions such as oxygen delivery, nutrient supplementation, and metabolic transport [44,45]. The directed migration and proliferation of keratinocytes and fibroblasts not only accelerate wound tissue formation but also synthesize collagen to provide mechanical support for the newly formed tissue [46]. Notably, angiogenesis exhibits synergistic effects with cell proliferation [47]. For instance, the upregulation of the growth factor EGF not only promotes cell activation and migration but also activates the formation of microvascular networks [48,49]. This creates a positive feedback loop that further accelerates the healing process of ideal wound surfaces.

At the same time, maintaining a stable physical microenvironment is equally essential for ideal wound healing. For example, maintaining an optimal level of moisture at the wound site promotes cell migration and controls metabolic exudate to reduce the risk of secondary infection [50,51]. Maintaining a local temperature around the wound that is close to the actual physiological range helps enhance tissue proliferation and blood perfusion [52]. Moreover, endogenous electric fields also play a crucial role in cell migration and tissue regeneration regulation [53,54,55]. Hence, simulating an electrically stimulated environment at an ideal physiological intensity can further promote cell migration and division around wounds, and the expression of related proteins [56,57].

Generally speaking, an ideal wound healing environment requires a delicate balance between inflammation regulation, cellular angiogenesis, antimicrobial protection, and physical stimulation. Based on this, an electronic skin system for wound healing should possess multimodal sensing and regulatory capabilities. By leveraging the synergistic effects of various signals, it constructs an efficient feedback mechanism and a dynamically adjustable microenvironment, thereby maximizing the attainment of an optimal wound healing state.

## 3. Biomimetic Thermotherapeutic Electronic Skins

During the dynamic process of wound healing, temperature serves as a physiological signal parameter that reflects inflammatory responses, blood flow changes, and tissue metabolic levels within the wound microenvironment [58,59,60,61]. Abnormal temperature elevation often indicates bacterial infection or exacerbated inflammation, while a decrease may suggest insufficient blood supply or tissue necrosis [62,63]. Therefore, highly sensitive, real-time temperature monitoring is crucial for evaluating wound healing. However, traditional heat patches are constrained by resolution, mechanical flexibility, and conformability, making continuous, precise surface temperature tracking challenging [64,65]. Thermoelectric polymer composite (TPC) and thermoregulation bionic electronic system (TRES) have emerged as promising biomimetic thermotherapeutic electronic skin materials, garnering increasing attention in recent years [66,67,68]. Research indicates that these materials not only possess biomimetic temperature sensing and regulation monitoring mechanisms [69,70], but also exhibit long-term stability and self-healing capabilities [71].

### 3.1. Electronic Skin Based on Thermoelectric Polymer Composite

**Figure 2:**
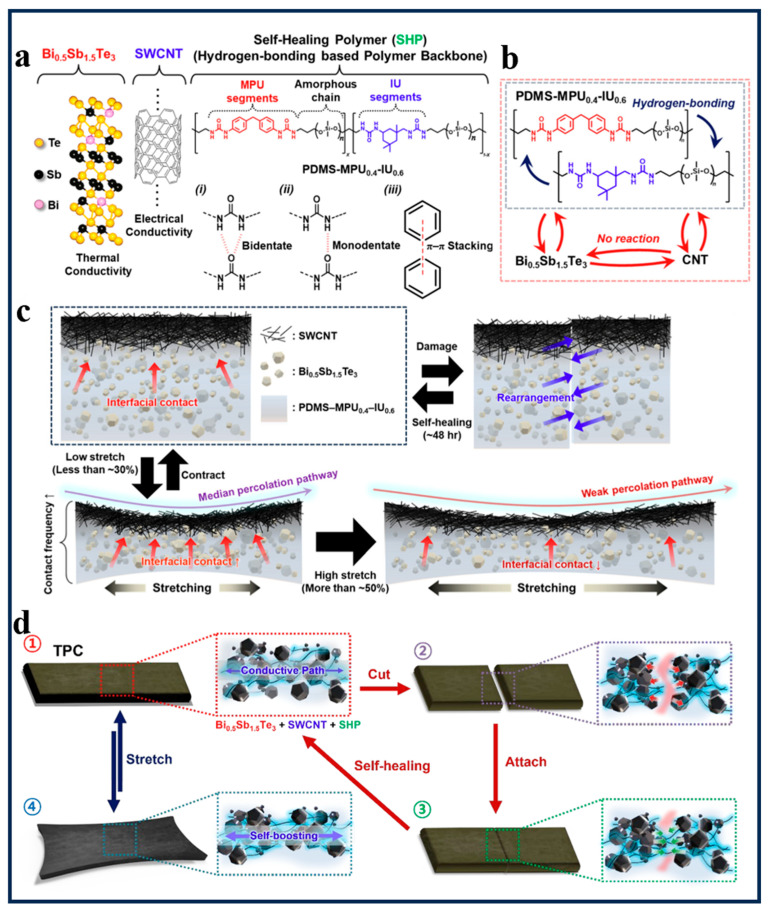
(**a**) Chemical structures and schematic illustrations of the precursors composed of Bi_0.5_Sb_1.5_Te_3_, SWCNTs, and a selfhealing polymer (SHP) with (i) bidentate, (ii) monodentate, and (iii) π−π hydrogen bonding interactions (**b**) Schematic of the mechanism involved in the formation of TPC via interaction between Bi_0.5_Sb_1.5_Te_3_, SWCNT, and SHP. (**c**) Microstructural configuration of the TPC during stretching (with electrical boosting) and self-healing. (**d**) Microstructural configuration of the TPC during stretch (Step 1 → 4 → 1) and self-healing (Step 1 → 2 → 3→ 1). (Adapted with permission [66], Copyright 2025, American Chemical Society).

Thermoelectric polymer composites represent an emerging material technology [72], characterized by high Seebeck coefficients, high sensitivity, and rapid response [73,74], with the ability to convert human body heat [75]. Here, Song’s group [66] developed an electronic skin based on thermoelectric polymer composites. This material integrates temperature sensing, self-healing, and stretchability, while its self-sufficient energy generation capability offers novel approaches for physiological temperature monitoring and active intervention-based intelligent wound healing systems [76]. Song’s group [66] first demonstrated that the development of self-healing materials (SSM) overcomes the limitations of electronic skin in terms of stretchability and self-repair [77]. while enhancing its stability and durability [78]. However, due to their lack of temperature-sensing capability, Song proposed the fabrication of a stretchable, self-healing, thermoelectric polymer composite (TPC) inspired by human skin. As shown in Figure 2a, the TPC comprises the thermoelectric material Bi_0.5_Sb_1.5_Te_3_, single-walled carbon nanotubes (SWCNTs), and a backbone polymer matrix PDMS-MPU_0.4_-IU_0.6_ composed of the self-healing polymer SHP. The PDMS-MPU_0.4_-IU_0.6_ backbone polymer matrix is dynamically reconfigured within the polyurea backbone through bidentate coordination, monodentate coordination, and π−π stacking interactions. Conductive fillers within the SHP matrix undergo orientation and rearrangement during mechanical deformation, forming a more continuous conductive network. This generates an “electrical boost” effect and enhances electrical properties under strain. This phenomenon arises from the strong hydrogen bonds within the polyurea structure. These bonds confer high electrical resistance to the material, while also increasing its absolute elastic modulus [79]. As shown in Figure 2b,c, during the dynamic structural evolution of TPC under tensile stress (steps 1 → 4), the progressive refinement of the percolation network and filler alignment enhances electrical conductivity and thermoelectric performance. In contrast, during the self-healing evolution (steps 1 → 2 → 3), these properties can be fully restored even after physical fracture. This design enables TPC to autonomously restore its original electrical and thermoelectric properties through molecular reconstruction, achieving self-healing of both structural integrity and conductive pathways. Notably, Song’s group [66] further analyzed the physicochemical properties of TPC. SHP retains its self-healing and dynamic reconstruction capabilities even after damage [80,81], As shown in Figure 2d, during the progressive formation of the TPC involving Bi_0.5_Sb_1.5_Te_3_, SWCNT, and SHP, arrows representing potential interactions between thermal conductive particles and the polymer matrix (red) and SHP self-healing (blue) indicate that the TPC exhibits polymer-dominated mechanical behavior during thermal cycling, demonstrating reversibility and thermochemical stability for thermoelectric applications. Concurrently, SHP/Bi_0.5_Sb_1.5_Te_3_ composites with ratios ranging from 1:1 to 1:6 were prepared to evaluate their mechanical properties.

**Figure 3:**
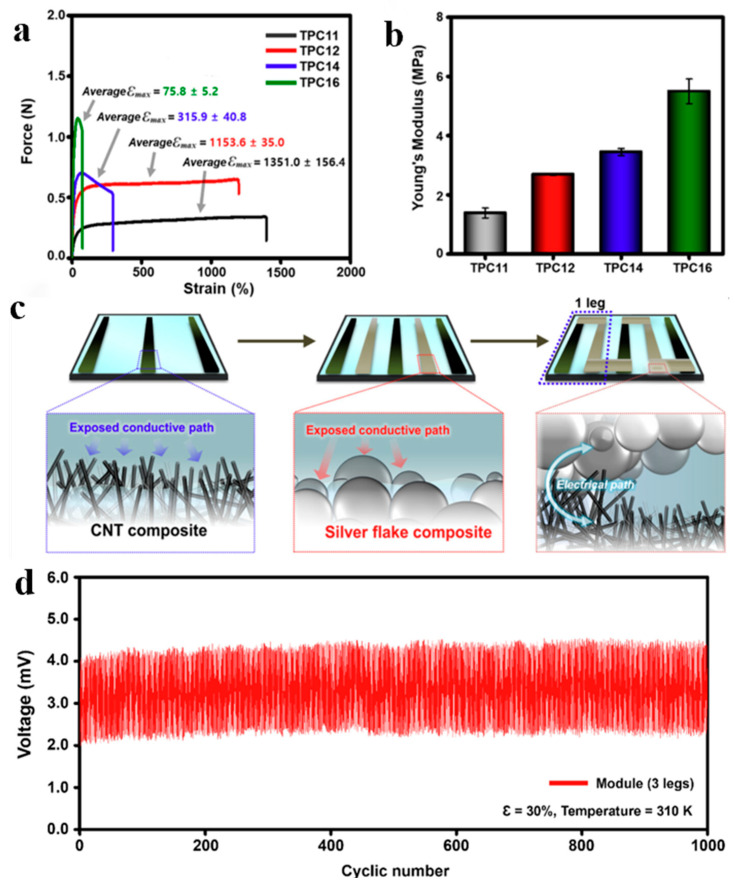
(**a**) Force per strain. (**b**) Young’s modulus of the TPC with various weight ratios of SHP and Bi_0.5_Sb_1.5_Te_3_ (SHP/Bi_0.5_Sb_1.5_Te_3_ = 1:1, 1:2, 1:4, and 1:6, respectively). (**c**) Module fabrication process (TPC/Ag-SHP/Ag-SHP module) and the corresponding electrical conduction pathways. (**d**) Voltage generation during cyclic stretching (30% strain, 1000 cycles). (Adapted with permission [66], Copyright 2025, American Chemical Society).

As shown in Figure 3a,b, under a constant elongation rate of 20 mm/min, TPC exhibits a maximum tear strain value of 1395% and a maximum toughness value of 5199.5 kJ/m^3^, outperforming the properties of the original MPU0.4-IU0.6 matrix [82], indicating that SHP/Bi_0.5_Sb_1.5_Te_3_ filler loading at ratios of 1:1 and 1:2 facilitates a favorable balance between mechanical robustness and tensile properties. As shown in Figure 3c,d, a functional modular system was successfully fabricated by combining TPC material and Ag-SHP in a 1:2 ratio. After 1000 cycles of repeated stretching, the mechanical stress exhibited remarkably stable performance, significantly enhancing the system’s scalability [83]. TPC generates voltage in response to temperature changes. Following the self-healing process under thermal measurement temperatures of 302–310 K, the conductivity remains largely unaffected by temperature variations. The Seebeck coefficient decreased from approximately 70 μV/K to 63 μV/K, while the power factor decreased from 2.19 × 10^−4^ μW/m·K^2^ to 2.02 × 10^−4^ μW/m·K^2^. This indicates that the non-recovery rate of the TPC’s thermoelectric properties is less than 10%. Song’s group attributes this to tensile deformation promoting orientation-dependent packing and densification of Bi_0.5_Sb_1.5_Te_3_ particles with SWCNTs within the SHP matrix. This facilitates the formation of more continuous and directionally aligned thermoelectric transport pathways, enhancing high-energy carrier transport. Compared to conventional self-healing systems, this electronic skin maintains highly stable comprehensive performance when exposed to moisture such as sweat and humidity [82], addressing the shortcomings in temperature control of existing technologies and advancing artificial skin development toward mimicking natural skin’s sensory capabilities. Future TPCs can further integrate highly polar self-healing polymer matrices with next-generation intelligent modular systems, offering enhanced solutions for expanding structural adaptability and dimensional scalability between temperature sensing and thermoelectric systems [84].

### 3.2. Electronic Skin Based on Interactive Thermoregulation Bionic Electronic System

As the largest organ in the human body, the skin possesses complex thermoregulatory functions [68,85]. Within healthy skin, thermoregulation is typically governed by the reciprocal control of neurovascular systems [86,87]. However, in injured areas, this regulatory mechanism becomes disrupted, often triggering inflammation and resulting in the loss of local temperature perception and regulation [88,89]. To address these challenges, traditional research has primarily focused on developing bionic temperature sensors for wearable devices. Nevertheless, most of these devices lack self-thermoregulatory capabilities [68,90,91].

**Figure 4:**
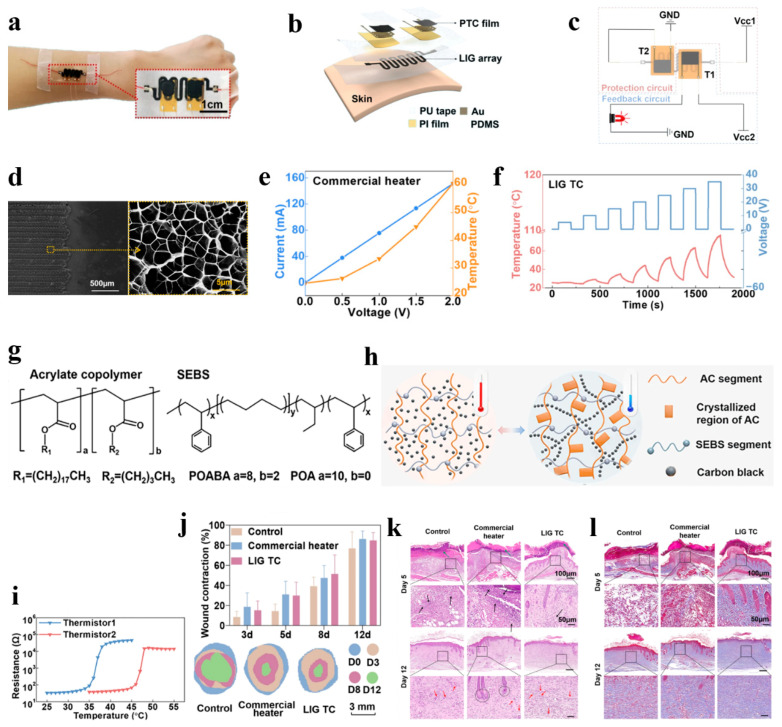
(**a**) The actual picture of TRES placed on the arm. (**b**) Temperature self-regulation mechanism of TRES. (**c**) Assembly diagram of TRES. (**d**) SEM image of LIG (texture structure and porous structure). (**e**) The relation between temperature and current of a commercial heater with voltage. (**f**) Joule thermal properties LIG TC. (**g**) Molecular formula of functional polymers. (**h**) Working mechanism of PTC thermistors. (**i**) Temperature response performance of PTC thermistors. (**j**) The percentages of wound contraction in 12 days and the changes in the wound boundaries. (**k**) Histological analysis of the wounds in commercial group treated via H&E staining and Masson staining (*n* = 3, *p* < 0.05). (**l**) Histological analysis of the wounds in TRES group treated via H&E staining and Masson staining (*n* = 3, *p* < 0.05). (Adapted with permission [67], Copyright 2024, Springer Nature).

In this context, as shown in Figure 4a, Geng’s group [67] proposed an electronic skin based on an interactive thermoregulation bionic electronic system (TRES). This system aims to replace the thermoregulatory function of damaged skin using external force influence, thereby maintaining the wound healing environment within a specific optimal temperature range. As shown in Figure 4b, compared to the electronic skin proposed by Li’s group [92] which consists of a polyolefin elastomer nanofiber membrane (POENM) with enhanced skin breathability and heat dissipation capabilities, and phase change materials (PCMs) that only exhibit limited temperature regulation within a controlled range [93,94], the TRES electronic skin system—comprising a laser-induced graphene (LIG) array equipped with PDMS and two highly sensitive, switchable polymer PTC transistors capable of real-time temperature monitoring—can directly transmit signals from temperature monitoring sensors to a heater attached to the wound surface for immediate temperature regulation. This system demonstrates dynamic temperature control capabilities to restore damaged wound temperatures. Additionally, it exhibits high adaptability to ambient temperature variations. The top of the TRES is sealed with polyurethane (PU) tape, securing the thermal control unit LIG between the temperature control unit and the heat control unit. As shown in Figure 4c, during the self-regulation process, the TRES achieves closed-loop feedback by relying on the thermally sensitive resistive state transition of the heat control resistors T1 and T2. When the heat control unit TC detects a low temperature, it triggers the high-resistance state of T1 to rapidly decrease, conducting the external feedback path to activate heating and sound an alarm. Conversely, exceeding the temperature threshold increases T2’s resistance, achieving automatic current limiting and cooling. Furthermore, as shown in Figure 4d, the CO_2_ laser process forms a graphene structure with porous and textured features in the LIG [95]. This endows the LIG with high electrical conductivity, excellent thermal conductivity, and mechanical flexibility, laying the foundation for constructing an effective adaptive temperature control unit. As shown in Figure 4e,f, approximately 40 °C is a suitable thermal stimulation temperature. Compared to commercial standard heaters, the current required to reach this temperature far exceeds safe human limits. When TIG TC reaches 40 °C, its power and current are only about 0.15 W and 4 mA, respectively. It exhibits shorter thermal response times, higher safety, and enhanced Joule heating effects. As shown in Figure 4g, the styrene–ethylene–butadiene–styrene (SEBS) molecular chains within the PTC and the acrylic copolymer (AC) with repeatable side chain crystallization contribute to the thermistor’s excellent response stability and repeatability [96]. The simulated conduction pathway is shown in Figure 4h. Above the melting point, the high viscosity of molten AC uniformly disperses carbon black CB within the PTC, resulting in high resistance. Conversely, when the temperature drops below the crystallization temperature, side-chain crystallization causes CB to aggregate in the amorphous regions, forming conductive pathways and reducing resistance. In the thermistor temperature response shown in Figure 4i, T1 and T2 effectively control the TRES temperature within the 35–45 °C range while accurately distinguishing specific values within this interval. Hence, it maximally satisfies the requirements for simulating natural temperature conditions. For systematic wound healing studies, Geng’s group utilized a mouse dorsal incision model. Comparative experiments were conducted using the TRES electronic skin against both a blank control and a commercially available heated dressing, as shown in Figure 4j–l. After 12 days, under appropriate temperature elevation provided by the TRES electronic skin, reduced inflammatory cell infiltration, increased collagen fiber deposition, and enhanced neovascularization were observed [97]. Compared with the control group, the overall healing rate was improved by approximately 10%. Economically, TRES offers the advantage of low cost and high efficiency. Notably, this electronic skin exhibits self-sensing, self-feedback, and self-stabilizing characteristics, overcoming the traditional “sensing-execution” separation model in conventional electronic skins [98]. Moving forward, it holds promise to integrate biodegradable substrate materials and multimodal intelligent algorithm fusion [99], thereby constructing a fully intelligent wound repair platform capable of real-time assessment and precise intervention. This advancement will propel the clinical translation of electronic skin in the field of regenerative medicine.

The core biological mechanisms for both research groups center on thermal and temperature regulation. Together, they demonstrate that biomimetic thermotherapeutic electronic skin offers efficient thermal sensing while suppressing inflammatory responses at wound sites through integrated thermostatic control. Simultaneously, it optimizes the pathways and environment for cell growth and migration, promoting the proliferation rate of new blood vessels near the wound site, thereby accelerating tissue repair at the targeted wound location. In addition, temperature serves as a crucial sensory variable. Through real-time feedback from the electronic skin system, it transmits immediate signals regarding local inflammatory states and tissue regeneration, providing dynamic monitoring information for the wound healing process.

## 4. Bioelectrically Active Electronic Skins

In recent years, wound therapies guided by electrical stimulation have been demonstrated to promote cell orientation and repair by reconstructing the endogenous electric field surrounding damaged tissues [7,100,101]. Nevertheless, with the global incidence of chronic wounds increasing annually, traditional electrical stimulation systems—relying on rigid electrodes and external power sources—largely suffer from insufficient flexibility, low signal coupling efficiency, and potential damage to surrounding soft tissues [102,103]. These limitations hinder the continuous and precise regulation of more complex physiological microenvironments [10]. Given this context, integrating flexible electronics with biomimetic wearable materials offers new opportunities for electronic skin in wound healing [104,105]. Recently, increasing evidence suggests that the skin’s intrinsic “endogenous electric field (EF)” is a key regulator of wound repair [106,107,108]. Additionally, multidimensional physiological parameters—including electrical and optical signals—play crucial roles in regulating cell migration, inflammatory responses, and angiogenesis during the restoration of wound microenvironment homeostasis [6,109,110,111]. Reconstructing this bioelectric microenvironment via electronic skin has emerged as a frontier in promoting tissue regeneration [112].

### 4.1. Electronic Skin Based on Multi-Responsive Sustained Drug-Delivery System

Melanoma is a highly invasive malignant tumor with a high incidence and unfavorable prognosis [113]. Current conventional treatments, such as chemotherapy, surgery, and photodynamic therapy, all face limitations, including restricted efficacy, insufficient penetration depth, or secondary wound infection [114,115,116]. Despite the potential curative capacity of immunotherapy, its clinical response rates remain suboptimal [117]. However, recent years have witnessed rapid advancements in nanomaterial-based drug delivery systems utilizing smart stimulus-responsive technologies [118,119]. Such systems enable precise, controllable drug release in response to external stimuli like heat, pH, light, or electricity, thereby enhancing the bioavailability and selectivity of anticancer drugs [120].

Upon this foundation, Zheng’s group [121] innovatively designed a multi-responsive controlled-release electronic skin (PADM-MX-Ag-Si@Dox) for drug delivery. PADM-MX-Ag-Si@Dox was constructed using porcine decellularized dermal matrix (PADM) as the substrate. Its overall structure comprises MXene nanosheets, silver nanowires (AgNWs), and multi-responsive TSOHSiO_2_@Dox microspheres. Compared to traditional natural hydrogel dressings, PADM, composed of natural collagen macro-nanofibers, provides an ECM scaffold structure mimicking the dermis, playing a crucial role in promoting cell adhesion, angiogenesis, and wound healing processes [122,123]. Compared to conventional patches, AgNWs and two-dimensional MXene are uniformly coated onto collagen nanotube surfaces modified with highly adsorbent PADM to form a continuous conductive network. This architecture endows the electronic skin with outstanding antibacterial properties and biocompatibility. In addition, PADM-MX-Ag-Si@Dox structures incorporating these nanomaterials exhibit a smoother surface compared to PADM-only structures while retaining the characteristic, well-ordered d-band structure typical of collagen fibers [124]. Building upon this structural advantage, PADM-MX-Ag-Si@Dox exhibits a tensile strength of approximately 22.76 MPa, enabling it to withstand tissue traction during wound healing while maintaining excellent mechanical stability [125]. The three-dimensional porous structure of the PADM provides a stable scaffold for microsphere anchoring and tissue cell growth, which enables the uniform distribution of TSOHSiO@Dox microspheres with high drug loading capacity throughout the electronic skin. This allows for the intelligent release of Dox under multiple signals, including temperature, pH, and electrical stimulation. Moreover, in the simulated temperature-responsive mechanism, the hydrophobic shrinkage effect of the P (NIPAM-co-AA) chains on the TSOHSiO_2_ surface further promotes Dox release. Concurrently, in the simulated pH-responsive mechanism, the TSOHSiO_2_@Dox microsphere volume contracts due to enhanced intermolecular electrostatic repulsion caused by deprotonation of -COOH groups, thereby releasing Dox. Under these conditions, Zheng’s group [121] demonstrated that microspheres achieve a maximum controlled-release efficiency of approximately 47–55% at 37 °C and pH 6.8. This exhibits their dynamic responsiveness and precise regulatory capacity for drug release, highlighting their potential for intelligent drug delivery applications [126]. Furthermore, due to insufficient interfacial bond strength, the negatively charged TSOHSiO_2_@Dox microspheres are loosely adsorbed onto collagen nanofibers. Under external electrical stimulation (ES), these microspheres peel off and are released as they migrate toward the anode. This phenomenon significantly enhances the controlled release efficiency of the composite electronic skin to 52.42%. At the cellular level, compared to the conventional microenvironment, the number of cells in the DNA synthesis and post-synthesis phases increased under ES. Furthermore, the significant reduction in forward and side scatter values indicates a smaller cell size. These findings collectively demonstrate that ES can synergistically activate ion channels, ROS, and growth factor expression by creating a conductive microenvironment, thereby enhancing cellular activity and proliferation potential [127,128]. Zheng’s group [121] also demonstrated that PADM-MX-Ag-Si@Dox possesses the capability to monitor melanoma lesion movement and postoperative wound healing in real time. Through multi-response drug release and ES synergy, this electronic skin effectively suppressed over 90% of melanoma recurrence while accelerating wound healing, establishing a forward-looking paradigm for postoperative melanoma management and wound repair. Overall, the core biological mechanism of this electronic skin lies in its ability to directly intervene in cellular activity by utilizing TSOHSiO_2_@Dox as a responsive unit. This unit synergistically drives targeted drug release through the ES while accelerating the migration rate of fibroblasts toward the wound site. Consequently, it creates a physicochemical environment conducive to angiogenesis and tissue reconstruction. In the future, efforts will continue to focus on clinical translational applications, driving the development of smart dressings toward greener and more intelligent directions. Such advancements aim to provide more cost-effective and environmentally friendly solutions for global tumor treatment and postoperative care [129,130].

### 4.2. Electronic Skin Based on Multifunctional Integrated Hydrogels

Over recent years, chronic wound healing has emerged as a major global health challenge, characterized by high infection risks, delayed healing, and a lack of effective monitoring methods [1,131]. Traditional wound dressings typically possess only single functions, lack mechanical compatibility with physiological tissues, and struggle to provide real-time environmental feedback in complex settings, hindering the realization of pathologically guided, responsive therapies [23]. Lately, functional hydrogel materials, with their outstanding biocompatibility and electroactivity [132,133], combined with electrical stimulation therapy, have gradually emerged as a new research direction for e-skin patches [134]. Accordingly, the research groups of Liu [135] and Shin [136] developed multi-modal responsive release electronic skin systems by integrating multi-hydrogel technologies with bioelectrically active materials. Liu’s research group [135] developed a multi-functional antibacterial electronic skin patch utilizing a tissue engineering scaffold. This electronic skin is primarily assembled layer-by-layer from a PDMS/PTFE membrane, liquid metal E-GaIn, and H_QPS_@MoS_2_ hydrogel. Based on the TENG, it achieves contact-separated wireless self-powering performance. Specifically, the PDMS/PTFE material enables the electronic skin to generate endogenous electric fields, which in turn accelerates wound cell proliferation. From a structural perspective, compared to conventional PDMS membranes, the PDMS/PTFE composite exhibits approximately 1.4 times higher mechanical strength due to its excellent interfacial compatibility. Furthermore, the presence of -CF_2_ groups in PTFE reduces surface energy, the hydrophobicity of PDMS/PTFE is significantly enhanced, improving sensing performance in humid environments and preventing bacterial regrowth interference in sweat. In addition to these chemical advantages, compared to conventional films, the PDMS/PTFE film exhibits a 3.8-fold increase in short-circuit current, demonstrating exceptional self-powering performance and the ability to release stable bioelectric signals through triboelectric sensing. With an average pore size of approximately 32.9 μm, the H_QPS_@MoS_2_ hydrogel exhibits an excellent porous structure that facilitates the absorption of wound exudate. It also possesses outstanding photothermal conversion efficiency of 44.38%, enabling it to generate a thermal effect under near-infrared stimulation and catalyze the production of ·OH radicals. Collectively, these properties confer enhanced mechanical strength, uniform dispersion, and significant antibacterial activity within the hydrogel. Furthermore, its dual temperature and strain sensing capabilities enable real-time monitoring of wound status. Moreover, the multiple effective attachments of HQPS@MoS2 facilitate electrical signal transfer to the wound site. This provides an efficient conductive pathway that accelerates angiogenesis and promotes cell migration. Through contact separation mode alone, the TENG generates a stable voltage of 7.8 V and 97.5 nA without an external power source, which enhances the expression of angiogenic factors. Notably, under strain conditions, the reduced carrier travel distance resulting from the thinner E-GaIn layer further enhances electron transport efficiency [137]. Meanwhile, Shin’s group [136] developed a bioelectrically active electronic skin based on multiple hydrogel materials. This electronic skin utilizes a PHEA hydrogel matrix as its substrate. A highly adhesive and tough PDA hydrogel facilitates tissue adhesion, while a PEDOT:PSS hydrogel with excellent electrochemical properties serves as the working electrode. Interconnections are achieved through Ag foil hydrogels. Building upon these functional capabilities, compared to conventional patches, the hydrogel-based electronic skin exhibits a low Young’s modulus and higher water content, enabling accelerated drug delivery and impedance isolation of the wound surface under ES [138,139]. Furthermore, compared to electronic skin without added electric field stimulation, wound closure rates improved by approximately 15% with ES stimulation therapy. This treatment also generated more skin appendages, increased granulation tissue thickness, and resulted in reduced inflammatory responses. Shin’s group [136] also innovatively designed a drug delivery strategy based on iontophoresis combined with electrostatic repulsion to transport epidermal growth factor (EGF), which carries negative charges and is capable of regulating cell proliferation, migration, and differentiation, from a hydrogel-based electronic skin to the wound site [140]. Concurrently, Shin’s group [136] employed a bioimpedance measurement system utilizing dual electrodes at different sites, enabling the electronic skin to function as a monitoring tool for wound healing. Overall, together they leverage the biomimetic properties of hydrogels to establish a stable tissue-device interface capable of responding to dynamic physiological changes [141,142]. They also integrate the therapeutic benefits of electrical stimulation, which promotes cell proliferation and differentiation at the wound site [143], while innovatively incorporating sensors for real-time, non-invasive wound monitoring. This integration not only enhances the clinical efficiency of chronic wound management but also pioneers a new personalized treatment model [144]. The former primarily expands into dual-signal early warning capabilities for temperature and strain, while the latter emphasizes the diagnostic-therapeutic closed-loop feedback achieved through ion release and impedance mapping. From the perspective of core biological mechanisms, both research teams utilized hydrogels as platforms for regulating dynamic physical fields, employing ES as the primary intervention and stimulation method. Leveraging the hydrogels’ exceptional conductivity and biocompatibility, they achieved precise modulation of immune responses. The electronic skin system suppresses the excessive expression of pro-inflammatory factors while preventing the persistence of chronic inflammation. It enhances the release of growth factors and improves hemodynamics in the wound area, delivering essential oxygen and nutrients to the injured site. All of this significantly boosts the efficiency and capacity of tissue repair. These multifunctional hydrogel electronic skin patches offer a revolutionary tool for chronic wound management, achieving over 80% accelerated healing and 95% antibacterial efficacy through integrated technologies. Their future development holds promise for addressing more complex and diverse wound healing challenges by combining biocompatibility optimization with programmable drug delivery systems [145,146].

### 4.3. Electronic Skin Based on Photoelectric Synergistic Stimulation

Most electronic skin systems currently under investigation rely on single stimulation modalities such as pure electrical stimulation, resulting in varying degrees of deficiency in synergistic effect mechanisms. Furthermore, material design and cellular-level response mechanisms remain imperfect [147,148,149]. At present, integrating light stimulation into the electroactive mechanisms of wound healing has gradually emerged as a research spotlight [150,151,152]. Here, compared to approaches by Liu’s group [135] and Shin’s group [136] that combine electrical stimulation with electronic skin for wound healing and monitoring, Tian’s group [101] and Wang’s group [153] offer a novel perspective to address this challenge: the development of innovative bioelectrically active electronic skin centered on optoelectronic synergy. By integrating photonic energy transfer with electrical stimulation signals, this approach not only effectively reprograms the intrinsic wound healing process but also resolves the disconnect between infection control and cellular activity regulation present in existing technologies [154,155].

**Figure 5:**
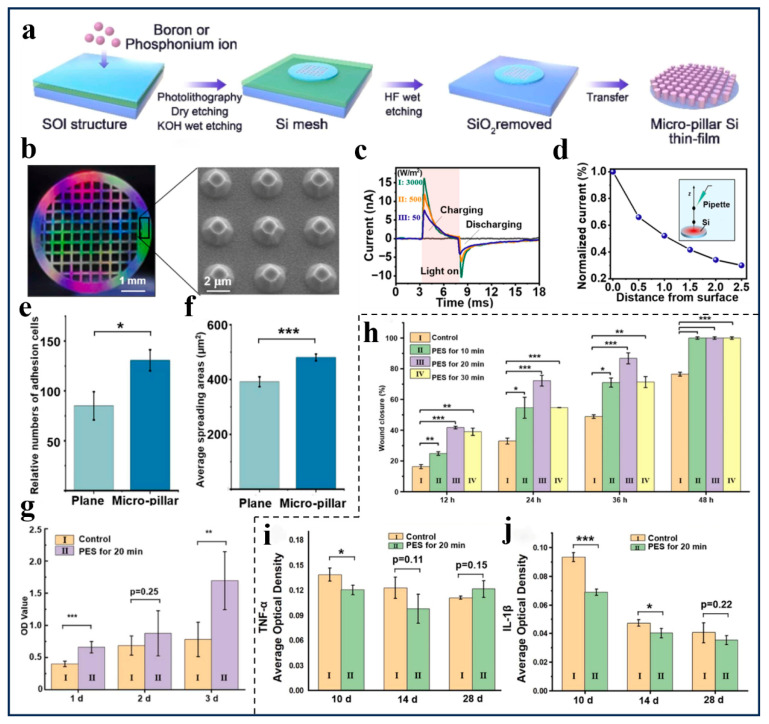
(**a**) Synthesis process of the Si-based photo-driven electronic skin. (**b**) Optical and SEM images of the p-type Si thin-film with micro-pillar 3D structures. (**c**) Photocurrent responses of p-type Si thin-films under different intensities of NIR irradiation. (**d**) Measured photocurrent as a function of vertical distance z in PBS solution. (**e**) The relative numbers of adhesion cells on the plane and micro-pillar p-type Si thin-film. (**f**) The average spearing areas of adhesion cells on the plane and micro-pillar p-type Si thin-film. (**g**) The proliferation ability of L929 cells with PES treatment for 20 min per day evaluated by CCK-8 assay, six in parallel. (**h**) Quantitative analysis of the wound closure ratio at 12, 24, 36 and 48 h after PES treatment for 10 min, 20 min and 30 min, respectively. (**i**,**j**) The average optical density (AOD) values of TNF-α and IL-1β in diabetic wound mice skin tissue with PES treatment at 10, 14 and 28d post surgery. * *p* < 0.05, ** *p* < 0.01, *** *p* < 0.001. (Adapted with permission [101], Copyright 2025, Elsevier Ltd.).

**Figure 6:**
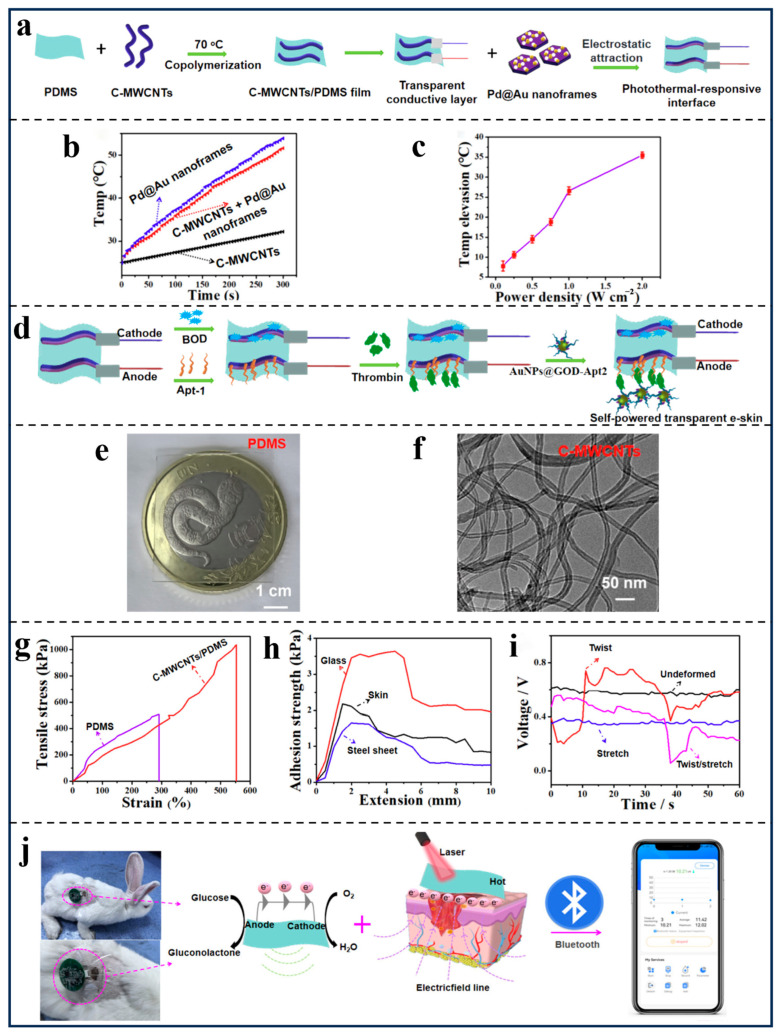
(**a**) Preparation Process of Transparent Conductive Layer and Photothermal-Responsive Interface. (**b**) The photothermal conversion absorption curves. (**c**) The relationship between the temperature of a C-MWCNTs + Pd@Au nanoframes (50 μg/mL) solution and the power density of NIR laser irradiation. (**d**) The Electrode Fabrications of the Self-Powered Transparent E-Skin. (**e**) Photographs of the PDMS films. (**f**) TEM image of C-MWCNTs. (**g**) Tensile-stress curves. (**h**) Adhesion strength of the C-MWCNTs/PDMS film on the surface of glass, skin, and a steel sheet, respectively. (**i**) The output voltage of the self-powered transparent e-skin with different film thicknesses. (**j**) Schematic illustration of the self-powered transparent e-skin. (Adapted with permission [153], Copyright 2025, American Chemical Society).

To address the challenge of healing refractory wounds, as shown in Figure 5a, Tian’s group [101] developed a light-driven electronic skin featuring a p-type silicon film with a three-dimensional microcolumn structure. This highly stable film is integrated with a near-infrared irradiation unit to enable its function. As illustrated in Figure 5b,c, the p-type silicon film leverages its superior photoelectric conversion properties to generate pulsed electrical signals under infrared irradiation. This process enables the regulation of physiological activity within the wound area. Moreover, the accumulation of cations at the interface between the p-type silicon film and PBS solution exhibits a positive correlation with irradiation intensity. As shown in Figure 5d, experiments were conducted by altering the perpendicular distance between the electrode film and the light plate. The results clearly demonstrate that at a distance of 1 mm, the photocurrent attenuation rate is only 50%, indicating that the photoconversion of the p-type silicon film exhibits millimeter-level high precision. Furthermore, as illustrated in Figure 5e,f, compared to flat-spread p-type silicon films, the microcolumn structure exhibits higher tissue adhesion. Through PES effects, it enhances intracellular calcium ion intensity by 1.23 times. Furthermore, this stimulation promotes fibroblast diffusion between the microcolumns. Since calcium ion channel levels are closely linked to cellular contractility [156,157], this demonstrates its strong potential in promoting cell regeneration. More notably, as shown in Figure 5g,h, compared to the control group without PES treatment, all PES-stimulated groups achieved 100% wound healing within two days, a 25% increase in healing rate. Additionally, 117% higher cell activity was observed after three days, confirming that combined photo-driven electrical stimulation promotes greater proliferation and migration of wound repair cells than a single stimulus alone. In an innovative application, Tian’s group [101] applied this electronic skin to diabetic wounds, as shown in Figure 5i,j. The PES stimulation therapy protocol applied to diabetic mice significantly suppressed the expression of pro-inflammatory factors IL-1β and TNF-α during the early inflammatory stage. During the early pathological phase, it effectively reduced pro-inflammatory levels and shortened the inflammatory cycle [158,159]. In addition, Tian’s group [101] further pointed out that upregulating the metabolic processes of the mTOR signaling pathway related to intracellular mRNA translation, together with enhancing actomyosin contractility through calcium influx, can be combined with the PES stimulation therapy based on electronic skin to jointly improve cell migration and wound repair capability. On the other hand, Wang’s group [153] proposed a transparent, self-powered electronic skin based on the synergistic interaction between endogenous electric fields and photothermal effects, featuring a three-layer structural design. On the other hand, Wang’s group [153] proposed a transparent, self-powered electronic skin based on the synergistic interaction between endogenous electric fields and photothermal effects, featuring a three-layer structural design. As shown in Figure 6a–c, the Pd@Au nanoframe with excellent photothermal conversion efficiency forms the photothermal responsive interface of this electronic skin. Compared to traditional single materials, the Pd@Au nanoframe significantly enhances the heating rate, achieving a temperature increase of 35.5 °C within 5 min under 808 nm, 1 W/cm^2^ near-infrared light irradiation. As shown in Figure 6d, the electrically stimulated sensing layer is composed of a glucose oxidase (GOD) catalytic reaction regulated by thrombin (TB), a protein capable of generating a stable intrinsic electric field. As shown in Figure 6e,f, the transparent conductive layer with over 90% transmittance features a composition of hexadecyltrimethylammonium bromide (CTAB)-functionalized multiwalled carbon nanotubes (C-MWCNTs), thermally deposited polydimethylsiloxane (PDMS). Moreover, as shown in Figure 6g,h, the cured transparent conductive layer C-MWCNTs/PDMS exhibits a uniaxial elongation rate as high as 550%, with an electronic skin adhesion strength of approximately 2 kPa. As demonstrated in Figure 6i, the output voltage of C-MWCNTs/PDMS remains largely unaffected by varying degrees of stretching and twisting. These properties confer high toughness and excellent, stable conductivity to the transparent conductive layer, establishing a robust environmental foundation for real-time visual monitoring of wound changes [160]. Notably, antimicrobial tests were conducted using Escherichia coli on the self-powered transparent electronic skin. With 0.59 V electrical stimulation, the system achieved a 198% increase in efficacy compared to the non-stimulated control group. Based on this, a synergistic photothermal/electrical stimulation platform was developed as shown in Figure 6j, enabling wireless collection and transmission of electrical signals and detection results via wireless devices. Also, Wang’s group [153] demonstrated that wounds covered with the self-powered transparent electronic skin—combining light-driven and electrical stimulation—healed completely in just 7 days, halving the 14-day healing time required for untreated traditional wounds. Overall, both research teams employed non-invasive interfaces to deliver controlled stimuli that mimic the skin’s bioelectric signals [161,162], ensuring real-time interaction within the wound microenvironment. Tian’s research group [101] focuses on light-driven and calcium-cascade mechanisms to achieve cellular-level responses to light and electricity [163]. Meanwhile, Wang’s group [153] emphasizes the synergistic effects between infrared hyperthermia and electrical stimulation. Furthermore, they integrated wound data acquisition and real-time monitoring functionalities directly into the conductive layer. From a core biological perspective, both research teams utilized light-responsive materials to convert external light energy into localized electrical signals. This generated a stable, controllable electric field within the electronic skin, guiding the migration of epithelial cells and fibroblasts toward the wound center. This process indirectly promoted tissue remodeling and matrix deposition. Additionally, optoelectronic couplers can also reduce the expression of pro-inflammatory factors and maintain the amplitude stability of internal physiological electrical signals, thereby improving the local steady-state environment while preventing tissue from suffering stimulus-induced damage. Looking ahead, both types of electronic skin have the potential to evolve from the concept of bioelectrically active electronic skin toward practical clinical applications. By integrating multimodal responses with photoelectric-synergistic mechanisms in drug delivery systems, they can establish more comprehensive regulatory mechanisms to develop personalized diagnostic and therapeutic plans for individual wounds [142,164,165].

## 5. Summary and Outlooks

In this work, we conduct a systematic review of recent innovations in electronic skin technology for wound healing, highlighting their respective advantages and potential applications. We focus on breakthroughs in two major technological pathways: biomimetic thermotherapy-type and bioelectrically active-type. Biomimetic thermotherapeutic electronic skins emphasize enhancing wound repair efficiency by simulating physiological thermal regulation processes. Among these, electronic skins based on TPC achieve dynamic adaptation of temperature and mechanical responses, while those based on TRES enable closed-loop thermal sensing and delivery. Bioelectrically active electronic skins focus on the connection between electrical signals and biological processes, encompassing multi-response drug delivery systems for on-demand administration, multifunctional hydrogel integrated platforms that simultaneously provide bioelectric stimulation, and solutions for photodynamic-electrical synergistic wound stimulation. Systematic analysis indicates that these technologies significantly accelerate vascular regeneration, collagen deposition, and re-epithelialization in chronic wounds by synergistically combining physical stimulation with the delivery of bioactive factors, establishing a new paradigm for regulating the wound microenvironment. Beyond this, these technologies simultaneously enhance both economic and social benefits. Economically, the adoption of self-powered systems eliminates the need for traditional battery replacements, reducing the energy costs of long-term therapeutic devices to near zero. Modular design serves as a pivotal interface between bioelectronics and clinical medicine; by encapsulating and integrating functional units, it significantly lowers overall maintenance, material replacement, and labor costs. Regarding social benefits, continuous electrical stimulation accelerates wound recovery and reconstruction. Combined with remote diagnostic systems, this approach breaks the physical isolation of patients with mobility limitations. Additionally, materials partially based on biodegradable components can automatically dissociate into environmentally harmless small molecules over time, substantially reducing the carbon footprint of medical devices and achieving an effect of “ecological healing.” Despite the continuous emergence of new electronic skin materials, their long-term biocompatibility requires further evaluation. Particularly under conditions of sustained thermal regulation or physical stimulation generated by electronic skin systems, issues such as inflammatory responses between material molecules and targeted wounds, risks of fibrous encapsulation, and cellular phenotypic characteristics need to be clarified. Additionally, the toxicological safety of the degradation products from biodegradable materials must also be verified through experimental testing. Meanwhile, in terms of engineering implementation, electronic skin is transitioning from laboratory prototypes to clinical products. However, this process continues to face significant challenges related to scaling and engineering conversion. This is reflected in the fact that most current healing systems rely on highly sophisticated microscale and nanoscale manufacturing. Consequently, these directional production methods lack scalable models capable of maintaining long-term stability. At the regulatory level, different types of electronic skin lack uniform physicochemical properties and healing mechanisms, potentially affecting product classification issues for medical devices and pharmaceuticals. This results in a more cumbersome and complex approval pathway and evaluation criteria for electronic skin compared to traditional dressings. Therefore, accelerating the engineering implementation and clinical validation of electronic skin, promoting its safe, efficient, and scalable development, and establishing a robust regulatory evaluation system will be crucial to overcoming existing bottlenecks and achieving its widespread application in precision wound diagnosis and treatment. Here, we outline the following outlook:

(1) Development of Long-lasting Biocompatible Materials. Although innovative materials have been effectively applied in electronic skin research and development, most current innovations primarily focus on enhancing existing technologies and diversifying design combinations, lacking further research into integration with biological tissues. Concerns persist regarding residual metallic components within materials and whether prolonged attachment of electronic skin materials may generate additional toxins. Therefore, future research could focus on breakthroughs in the long-term coexistence mechanisms between electronic components and biological tissues. For instance, by developing gradient-degradable electrode materials and establishing in vivo metabolic databases for bioelectrically active materials, the compatibility of new materials can be continuously monitored and evaluated.

(2) Innovation in Multi-Module System Integration. While current electronic skin research has achieved significant breakthroughs in modular sensing accuracy and power supply technologies, system integration still faces resource utilization bottlenecks. For instance, the photoelectric conversion efficiency of optoelectronic hybrid devices is constrained by the light transmittance of flexible substrates. Similarly, although TRES enable dynamic temperature regulation, their potentially excessive power consumption may render them incompatible with portable wearable modules. Therefore, researchers can achieve system-level energy efficiency optimization by combining multi-modal energy harvesting from external sources with intelligent coordination of lightweight energy storage units. Concurrently, optimizing device structural design, establishing AI large-model databases for localized deployment, and enhancing personalized energy conversion efficiency are also essential.

(3) Breakthroughs in Scalable Manufacturing Technologies. While most innovative wound-healing electronic skin materials currently employ low-cost production processes, challenges persist in the design complexity and manufacturing reliability of core modules. For instance, TPC electrode array etching and hydrogel microfluidic channels remain difficult to replicate in low-cost, high-volume production. Moving forward, development feasibility can be further integrated by incorporating cost and complexity into comprehensive evaluations. This approach will enable the reconstruction of manufacturing-oriented collaborative design, anchoring a strategic balance between universal applicability and reliability.

(4) Reform of Underlying Mechanisms. While current technologies can systematically detect and repair wounds through physical responses such as electro-optical-thermal interactions, these largely remain within traditional stimulation paradigms. Future breakthroughs could focus on establishing feedback loops based on electroactive hydrogels, driven by underlying mechanisms such as immune-electrical signal interactions. These systems would release electric fields triggered by specific bioelectric signals to regulate wound cell activation and drug delivery mechanisms, thereby constructing closed-loop repair systems integrating sensing, intervention, and feedback.

(5) Clinical Translation-Driven Approach. Future researchers can continue leveraging the studied electronic skin samples to advance the establishment of standardized animal models, constructing a unified dynamic evaluation system that balances safety and innovative therapeutic efficacy, and refining the review process and regulatory framework for new electronic skin products entering the market. Furthermore, a collaborative mechanism involving academia, industry, and healthcare institutions can be established to foster interdisciplinary training encompassing material design, system integration, and pathological research. This will propel novel electronic skin systems toward the ultimate goal of clinical implementation as modern medical wound treatment solutions.

In summary, as wound healing remains a hotly debated topic in contemporary medical research, and emerging electronic skin technologies offer more forward-looking and scalable prospects for wound treatment, this review comprehensively summarizes research progress in biomimetic thermotherapeutic and bioelectrically active electronic skin technologies. It provides an in-depth analysis of their structural advantages and potential applications. Finally, it provides an outlook for the future development of electronic skin in wound healing. We sincerely hope this review offers valuable insights to researchers.

## Data Availability

No new data were created or analyzed in this study.
